# System biology approaches identified novel biomarkers and their signaling pathways involved in renal cell carcinoma with different human diseases

**DOI:** 10.1042/BSR20221108

**Published:** 2022-11-21

**Authors:** Md. Saddam Hossen, Abdus Samad, Foysal Ahammad, Gabriel B.K. Sasa, Zhenggang Jiang, Xianfeng Ding

**Affiliations:** 1 College of Life Sciences and Medicine, Zhejiang Sci-Tech University, 310018 Hangzhou, People’s Republic of China; 2 Laboratory of Computational Biology, Biological Solution Centre (BioSol Centre), Jashore 7408, Bangladesh; 3 Department of Genetic Engineering and Biotechnology, Faculty of Biological Science, Jashore University of Science and Technology, Jashore 7408, Bangladesh; 4 Department of Biological Sciences, Faculty of Science, King Abdul-Aziz University, Jeddah 21589, Saudi Arabia; 5 Division of Biological and Biomedical Sciences (BBS), College of Health & Life Sciences (CHLS), Hamad Bin Khalifa University (HBKU), Doha 34110, Qatar; 6 Department of Science Research and Information Management, Zhejiang Provincial Centers for Disease Control and Prevention, Hangzhou 310051, China

**Keywords:** Functional enrichment, PPI and Regulatory biomolecules, Renal cell carcinoma, Survival analysis, System biology, Transcriptional profile and Comorbidity

## Abstract

Renal cell carcinoma (RCC) is a type of cancer that develops in the renal epithelium of the kidney. It is responsible for approximately 3% of adult malignancies, and 90–95% of neoplasms originate from the kidney. Advances in tumor diagnosis, innovative immune therapeutics, and checkpoint inhibitors-based treatment options improved the survival rate of patients with RCC accompanied by different risk factors. RCC patients with diabetes, hepatitis C virus (HCV), or obesity (OB) may have a comorbidity, and finding the risk factor for better clinical treatment is an urgent issue. Therefore, the study focused on network-based gene expression analysis approaches to learning the impact of RCC on other comorbidities associated with the disease. The study found critical genetic factors and signal transduction pathways that share pathophysiology and commonly use dysregulated genes of the illness. Initially, the study identified 385 up-regulated genes and 338 down-regulated genes involved with RCC. OB, chronic kidney disease (CKD), type 2 diabetes (T2D), and HCV significantly shared 28, 14, 5, and 3 genes, respectively. RCC shared one down-regulated gene versican (VCAN) with OB and HCV and one down-regulated gene oxidase homolog 2 (LOXL2) with OB and CKD. Interestingly, most of the shared pathways were linked with metabolism. The study also identified six prospective biomarkers, signaling pathways, and numerous critical regulatory and associated drug candidates for the disease. We believe that the discovery will help explain these diseases’ complicated interplay and aid in developing novel therapeutic targets and drug candidates.

## Introduction

Renal cell carcinoma (RCC) appears to be the most common type of kidney cancer, with over 90% of occurrences [1]. It is a sophisticated carcinoma that accounts for around 2% of all cancer diagnoses and deaths globally and is expected to rise in prevalence [2]. The prevalence of RCC varies significantly across the globe, with the highest rates found in North America and the Czech Republic [3]. Every year, 64000 new infections of RCC are recognized in the United States, and 14000 people die as a result of RCC [4]. It affects men more than women (ratio 1.7:1), and most of those affected are older, with an average age of 64 years [5]. When two or more coexisting diseases influence one other due to their shared pathogenesis, they are considered comorbidities [6]. Obesity (OB), hypertension, chronic kidney disease (CKD), and type 2 diabetes (T2D) represent established risk factors for RCC [7–9]. Their connections may contribute to the identification of new risk variables that could aid in the diagnosis and management of high-risk groups. Although these comorbidities are correlated to the prevalence of RCC, more research into the disease-modifying processes is recommended [6].

Recent research suggests that metabolic changes are essential in RCC biology, a tumor resistant to traditional chemo- and radiotherapy [10]. The metabolism of RCC cells creates a unique tumor susceptibility environment [11]. The metabolic system can be considered a central homeostatic process, and its dysfunction can result in several chronic metabolic diseases, including T2D, OB, and cardiovascular disease [12]. A massive amount of data have been analyzed extensively in recent decades to study the impact of OB/BMI on the prevalence of RCC. As a result, both men and women have been found to have a substantial association in terms of carcinogenesis [13,14]. OB is a chronic noncommunicable condition that affects almost a third of the world’s population [15]. OB is the second leading cause of cancer after smoking, according to cancer prevention experts [16]. OB is linked to a higher risk of metabolic illnesses like insulin resistance, T2D, dyslipidemia, nonalcoholic fatty liver disease, and specific cancers like RCC, according to growing evidence [17]. However, the connection between OB and RCC is still a mystery at all stages of the disease [18]. OB is responsible for 40% of all cancer fatalities in the United States, and a higher risk of OB-induced mortality has been demonstrated for many cancers, including RCC [19].

CKD patients may be highly susceptible to RCC, which has become a rise in recent years [20]. Significantly, when kidney disease develops in the environment of RCC, mortality rises dramatically, with patients frequently dying of a noncancer-related kidney disease consequence [21]. Furthermore, severe CKD was connected to a greater risk of RCC in individuals having end-stage renal disease [22]. RCC and CKD are associated, with 26–44% of RCC patients having CKD at the time of diagnosis [23]. Research demonstrated that 26% of kidney cancer patients had CKD before tumor nephrectomy, according to the Modification of Diet in Renal Disease equation [24]. The significant burden and harmful effects of CKD in renal cancer patients have primarily escaped the medical community’s notice [25]. T2D is a metabolic condition defined by hyperglycemia in the presence of decreased insulin sensitivity and insulin level [1] that affects millions of individuals worldwide. Because hypertension and OB are major RCC health issues closely associated with T2D, taking these factors into account when assessing the link between T2D and RCC is crucial [3]. T2D was linked to a higher risk of RCC [26].

T2D was related to a 60% higher risk for RCC in women in the Nurses’ Health Study, according to earlier research (NHS) [27]. Although T2D is linked to an elevated risk of a variety of cancers, its connection to RCC is unclear [28]. Furthermore, hepatitis C virus (HCV) infection is a major public health concern. HCV infection increases the risk of chronic renal disease, and multiple studies have linked it to RCC, cancer with a fast-rising global incidence [29,30]. HCV is nearly three times as prevalent in RCC patients than in the average population of Brazil [31]. Hence, genetic factors that are induced by RCC and result in a poor prognosis must be recognized.

**Figure 1:**
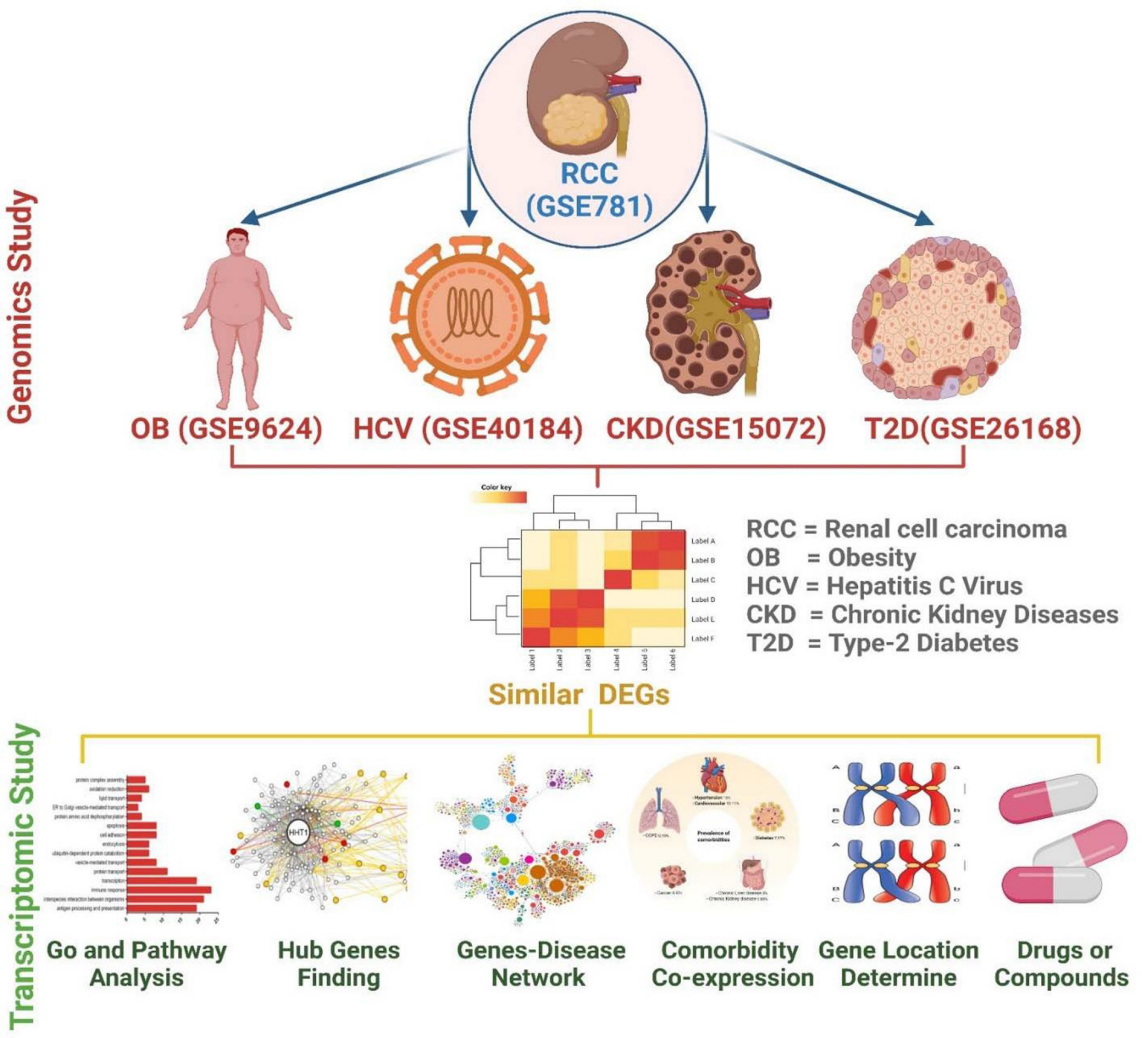
Graphical representation of the overall work performed in the present study Herein, the genomic study was performed initially in work; subsequently, conducted the transcriptomic research.

Various biomarkers and comorbidities were discovered to be implicated in the disease progression. This research aims to find the molecular mechanisms of the association of RCC and the four key comorbidities (OB, CKD, T2D, and HCV) and the pathways of their shared pathogenesis (Figure 1). Molecular enrichment of its common source will give a disease-modifying approach that may relate these comorbidities to RCC advancement.

## Materials and methods

### Dataset collection and normalization

To investigate the impact of RCC, we gathered and processed significant microarray datasets from the National Center for Biotechnology Information (NCBI) Gene Expression Omnibus (http://www.ncbi.nlm.nih.gov/geo/) and its genetic relationship with other prevalent diseases. Data normalization is critical in assessing experimental observations [2]. We analyzed the RCC gene expression data and four relevant conditions: OB, CKD, T2D, and HCV. A total of five datasets with accession numbers, including GSE781, GSE9624, GSE15072, GSE26168, and GSE40184, were used in the present study [32–36]. The RCC dataset (GSE781) is an Affymetrix Human Genome U133 Array [HG-U133] on the platform of GPL96 that was selected with nine case samples and eight normal samples. The Obesity microarray dataset (GSE9624) was established using the Affymetrix Human Genome U133 Plus 2.0 Array GPL570 (HG-U133 Plus 2), which contains five obese and six normal-weight children’s omental adipose tissue samples. The CKD dataset (GSE15072) is an [HG-U133A] Affymetrix Human Genome U133A Array on the GPL96 platform that includes nine CKD patients’ peripheral blood mononuclear cells, 12 hemodialysis patients’ samples, and eight healthy samples. The T2D (GSE26168) gene expression profile was created using an Illumina Human Ref-8 v3.0 expression bead chip on the GPL6883 platform with nine T2D peripheral blood samples patients and eight normal people. Finally, the HCV dataset (GSE40184) is a transcriptomic profile that was created using an [HG-U133A] Affymetrix Human Genome U133A Array from whole blood samples of ten HCV patients and eight healthy volunteers as controls, details are shown in Table 1. To identify and quantify the possible molecular mechanisms of human disorders, we used freely accessible gene expression microarray datasets. Limma R package has been used to discover differentially expressed genes (DEGs) in each dataset, following two factors: log2FC and *P*-value. For up-regulated genes, we used log2FC ≥ 2; for down-regulated genes, we used log2FC ≤ −2. Furthermore, significant DEGs were filtered out using a *P*-value<0.05.

**Table 1:** Details of the dataset, sample number, and platform used in the present study, along with the year and origin country

Disease	Identifier	Platform	Case vs Normal	Country	Year
RCC	GSE781	GPL96	9 vs 8	U.S.A.	2018
CKD	GSE15072	GPL96	21 vs 8	Italy	2020
T2D	GSE26168	GPL6883	9 vs 8	Singapore	2017
OB	GSE9624	GPL570	5 vs 6	Spain	2019
HCV	GSE40184	GPL96	10 vs 8	U.S.A.	2018

### Assessment of overlapping genes and their expression profile

Then, using the Venny v2.1 [37], we compared the RCC dataset with four additional disorders to find shared DEGs. A volcano plot made with ImageGP (http://www.ehbio.com/ImageGP/) was used to display all major DEGs. Bipartite graph theory [38] was employed to generate the gene-disease Network (GDN), which was then displayed via Cytoscape v3.7 [39].

### Location of chromosomes and tissues and disease association with genes

Understanding the pathophysiology of certain genes and identifying therapeutic targets require knowledge of their chromosomal position and expressional pattern. As a result, the DEGs’ chromosomal location was predicted using the ShinyGO web tool [40]. Furthermore, DEGs are distributed differently in different tissues, we used the PaGenBase dataset via the Metascape web server [41]. We analyzed the comorbidity profiles with the shared DEGs using the Metascape online tool and the *P*-value was set ≤0.05. The Expression Atlas database was used to analyze the expression pattern of the shared DEGs in other diseases [42]. The data on coexpression were collected using the log2FC from every gene with disease versus control datasets accounted. Applying the Morpheus online tool (https://software.broadinstitute.org/morpheus/), a clustered heat map had constructed using the disorder expression value (log2FC) for common DEGs.

### Functional strategy of genes

Enrichment analysis is a statistical and analytical tool for determining whether several genes contain statistical significance in diverse biological situations [43]. The GO resources contain structural and computational information about gene product-based functions [44]. In the presesnt study, we used the Enrichr web tool [45] to predict pathways and gene ontologies connected to DEGs using multiple databases. For cell informative pathways, we utilized the WikiPathway 2021 human, KEGG 2021 human pathway, Elsevier pathway collection, and Reac-tome (2016), whereas we regarded biological process (2021), cellular component (2021), and molecular function (2021) for gene ontologies with a fold‐change ≥2 and a *P*‐value cutoff of <0.05 was defined as statistically significant. Additionally, -log10(P) was utilized to quantify the importance of pathway associations and the enrichment of every GO terms by gene expression profiles [3]. ImageGP tool (https://www.ehbio.com/ImageGP/) has been used to visualize the enrichment plots.

### Analysis of protein–protein interaction

The investigation of protein interactions is the first stage in drug development and bioinformatics [46], providing a lot of information on how proteins work. The detailed analysis of protein–protein interaction (PPI) networks [47] is used to determine the number of complex biological processes. To discover the molecular methods of critical signaling pathways and cellular functions, PPI of common DEGs was constructed employing the STRING dataset through the NetworkAnalyst v3.0 [48]. The PPI network was created with the general PPI setting, *H. sapiens* as the organism, STRING with scientific proof as to the database, and a confidence score threshold of 900. After that, we analyzed the accuracy and concluded that the most likely hubs were the common nodes. Afterward, we used the cytoHubba plugin [49] through Cytoscape v3.7 [37] to discover possible hubs within the PPI network. Degree, maximal clique centrality (MCC), and edge-percolated component (EPC) were evaluated in the present study [49]. After that, we made comparisons and determined that the standard networks were the most likely hubs. Lastly, Cytoscape v3.7 was used to customize the networks.

### Identification of regulatory biomolecules

Significant variations in transcription and expression outcomes are caused by regulatory molecules, including transcription factors (TFs) and micro-RNAs (miRNAs). Because of their high stability and ease of detection in biological fluids, miRNAs have a lot of potential as biomarkers [4]. According to a bioinformatics study, a single miRNA can affect hundreds of target genes. Recently, abnormal miRNA expression in many cancers has been documented, implying that miRNAs can act as tumor suppressors and oncogenes [5]. As a result, we used the NetworkAnalyst v3.0 website [48] to predict TF-gene and miRNA-gene connections using experimentally verified JASPAR [50] and miRTarbase v8.0 [51] datasets. We selected all TF-gene and miRNA-gene interactions having degree distribution of 5 and 2, respectively, to exclude nonmajor signature molecules. Cytoscape v3.7 [39] was used to customize both networks. NetworkAnalyst has become an essential computational tool as the demand for gene expression-based statistics develops [52].

### Protein connecting with potential drugs

The analysis of protein–drug interactions (PDI) is critical for understanding the fundamental properties of ligand affinity. The identification of prospective drug molecules is one of the key goals of this type of research. Employing the NetworkAnalyst v3.0 website and DrugBank v5.0, numerous drug molecules are suggested based on shared DEGs and we used the Cytoscape v3.7 [37] for downloading and customizing the network data.

### Results of the study

**Figure 2:**
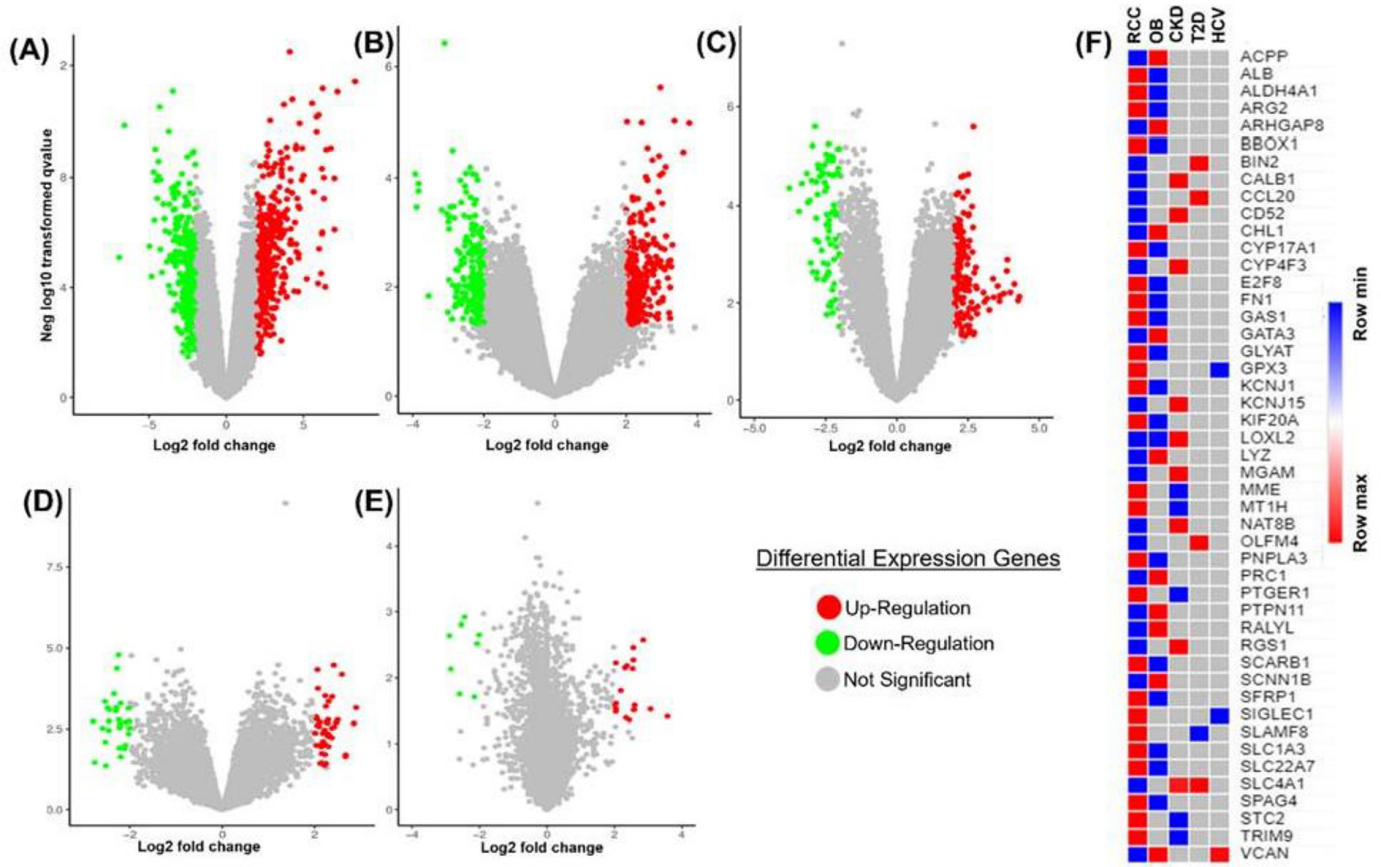
Volcano plot and heat map of all significant DEGs identified in the present study DEG distribution volcano maps in GSE781 (**A**), GSE9624 (**B**), GSE15072 (**C**), GSE26168 (**D**), and GSE40184 (**E**). The overexpressed and underexpressed DEGs are shown by red and green dots, respectively. (**F**) The heat map depicts the link between RCC and other associated genes in terms of log fold-change values.

We analyzed the RCC gene expression data and four relevant diseases including OB, CKD, T2D, and HCV. There are a total of five datasets with accession numbers including GSE781, GSE9624, GSE15072, GSE26168, and GSE40184 were used in the present study. Each dataset’s DEGs have been established, and several overlapping DEGs have been discovered. In RCC, OB, CKD, T2D, and HCV, we found 603, 478, 210, 75, and 24 significant DEGs (*P*<0.05), respectively (Figure 2A–E). The number of up-regulated genes was 314, 274 147, 46, and 18 among them, while the number of down-regulated genes was 289, 206, 63, 29, and 6 among them (Supplementary Tables S1–S5). We also created heat maps to display the relationship among the overlapping DEGs. The heat map in Figure 2F shows the relationship between genes in terms of log fold-change values.

### Differentially expression and distribution of DEG

**Figure 3:**
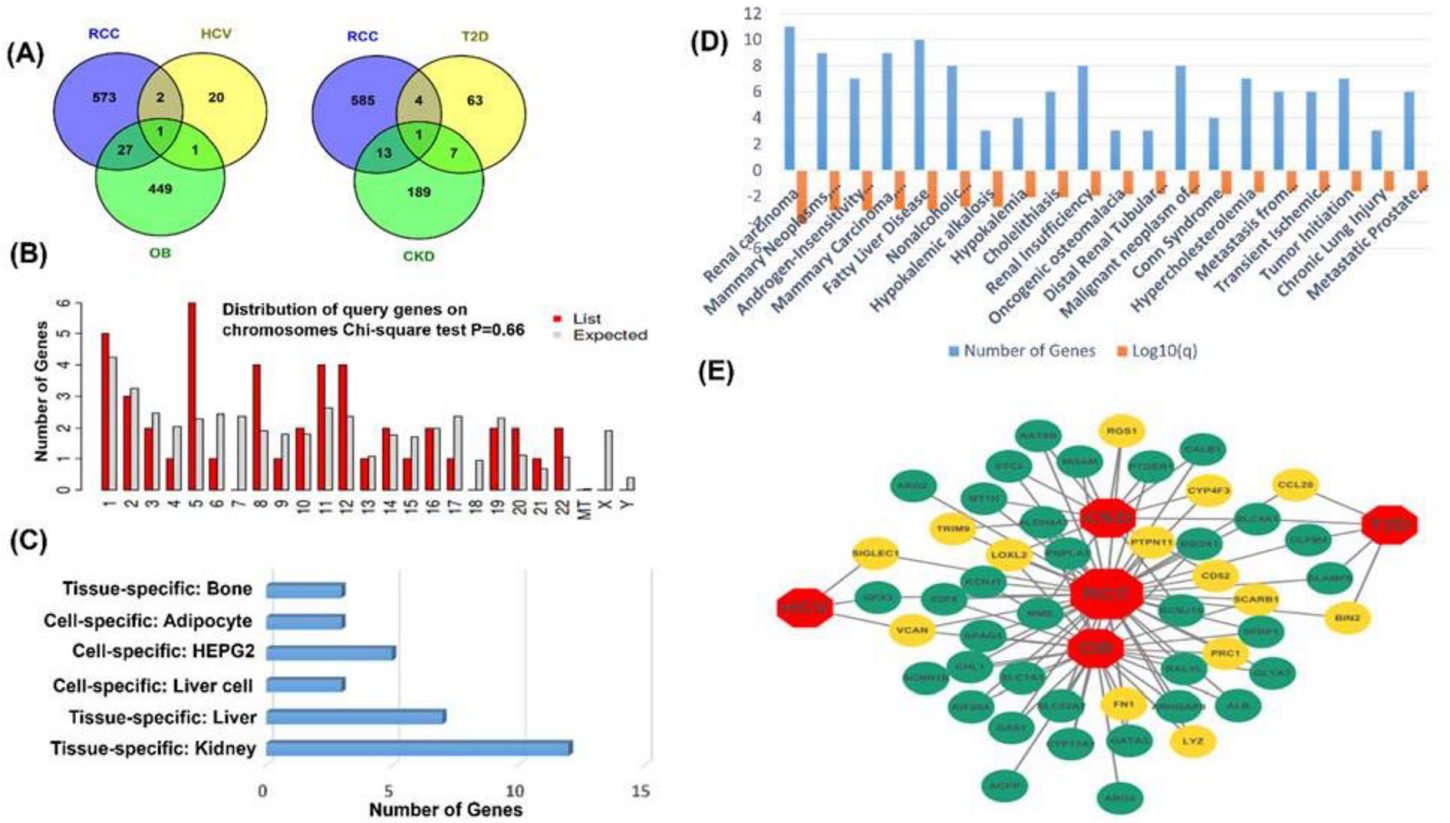
Shared dysregulated genes: a comparison, distribution, and comorbidities (**A**) Venn diagram comparison of gene expression: (i) RCC with HCV and OB, (ii) RCC with T2D, and CKD, (**B**) chromosomal location of genes, (**C**) tissue- and cell-specific distribution of genes, (**D**) top-20 diseases associated with shared DEGs, and (**E**) infectome–diseasome network where the octagon-shaped nodes represent five diseases, while circular nodes delineate up-regulated genes are represented by viridian green nodes, while yellow nodes represent down-regulated genes.

After comparing the RCC to other datasets, we discovered 47 unique shared DEGs, 16 of which were up-regulated and 31 of which were down-regulated (Supplementary Table S6). We used a cross-comparative study of gene expression profiles to better understand the pathogenic role of RCC in the mentioned diseases. The Venn diagram of Figure 3A shows that RCC shares 14, 5, 3, and 28 genes with CKD, DT-2, OB, and HCV. We developed a gene-disease relationship network (GDN) centered on the RCC to visualize their relationship as shown in Figure 3E. Remarkably, one gene (*VCAN*) was down-regulated among RCC, OB, and HCV, while a single gene *SLC4A1* was common among RCC, CKD, and T2D.

Identifying a protein at the transcriptional level necessitates knowledge of the genes’ exact cellular and molecular locations as depicted in Figure 3B,C. The majority of the common DEGs (six) were found on chromosome 5, with five DEGs on chromosome 1 and four DEGs on chromosomes 8, 11, and 12. Except for 7, 18, X, and Y chromosomes, the rest are evenly distributed throughout the genome. Shared DEGs were also missing in the

(Figure 3B). The majority of DEGs (12) are expressed in kidney tissue, followed by liver (seven) and HEPG2 (five), while the smallest number of genes (three) are expressed in liver cells, bone marrow tissue, and adipocyte cells (Figure 3C; Supplementary Table S7).

### Expressions of DEGs in other diseases and comorbidities

We selected the top 20 highly relevant diseases for our shared DEGs using the analysis of gene–disease corellation (Supplementary Table S8). Figure 3D shows the number of genes linked to common complications. The majority of the shared DEGs revealed in the present study were related to the development of renal carcinoma (11), followed by fatty liver disease (ten), mammary neoplasms (nine), mammary carcinoma (nine), and other diseases. Eight DEGs were discovered to be involved with nonalcoholic steatohepatitis, renal insufficiency, and malignant neoplasm of the kidney (Figure 3D).

**Figure 4:**
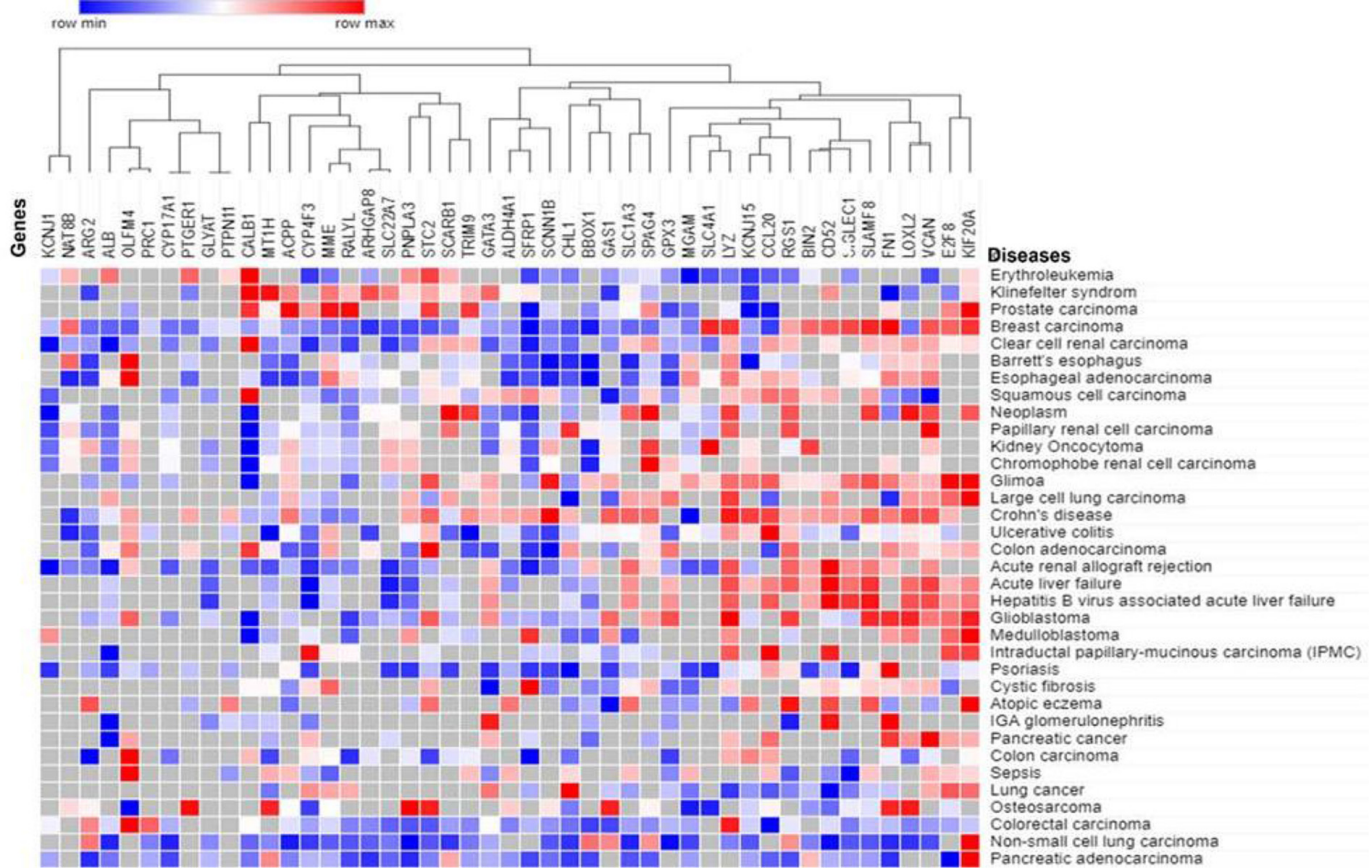
In diverse disorders, a heat map depicting the expression of common DEGs The clustering feature displays the associated coexpression of the genes based on the log2FC values of the shared DEGs, while the green- and pink-colored boxes represent over- and underexpression of the genes in respective disorders, respectively.

The Expression Atlas included coexpression data for 47 genes out of 47 shared DEGs for 35 health conditions, as shown in Figure 4 and listed in Supplementary Table S9. We discovered that the expression of common DEGs varies with diseases based on the heat map. As demonstrated by the pink-colored cluster, most DEGs were positively regulated in hepatitis B virus-associated acute liver failure, acute liver failure, glioma, neoplasm, glioblastoma, Crohn’s disease, prostate cancer, and so on in Figure 4. Conversely, as demonstrated by the green-clustered DEGs, DEGs were negatively regulated in non-small cell lung carcinoma, psoriasis, acute renal allograft rejection, esophageal adenocarcinoma, Barrett’s esophagus, pancreatic adenocarcinoma, etc. (Figure 4).

### Enrichment analysis

**Figure 5:**
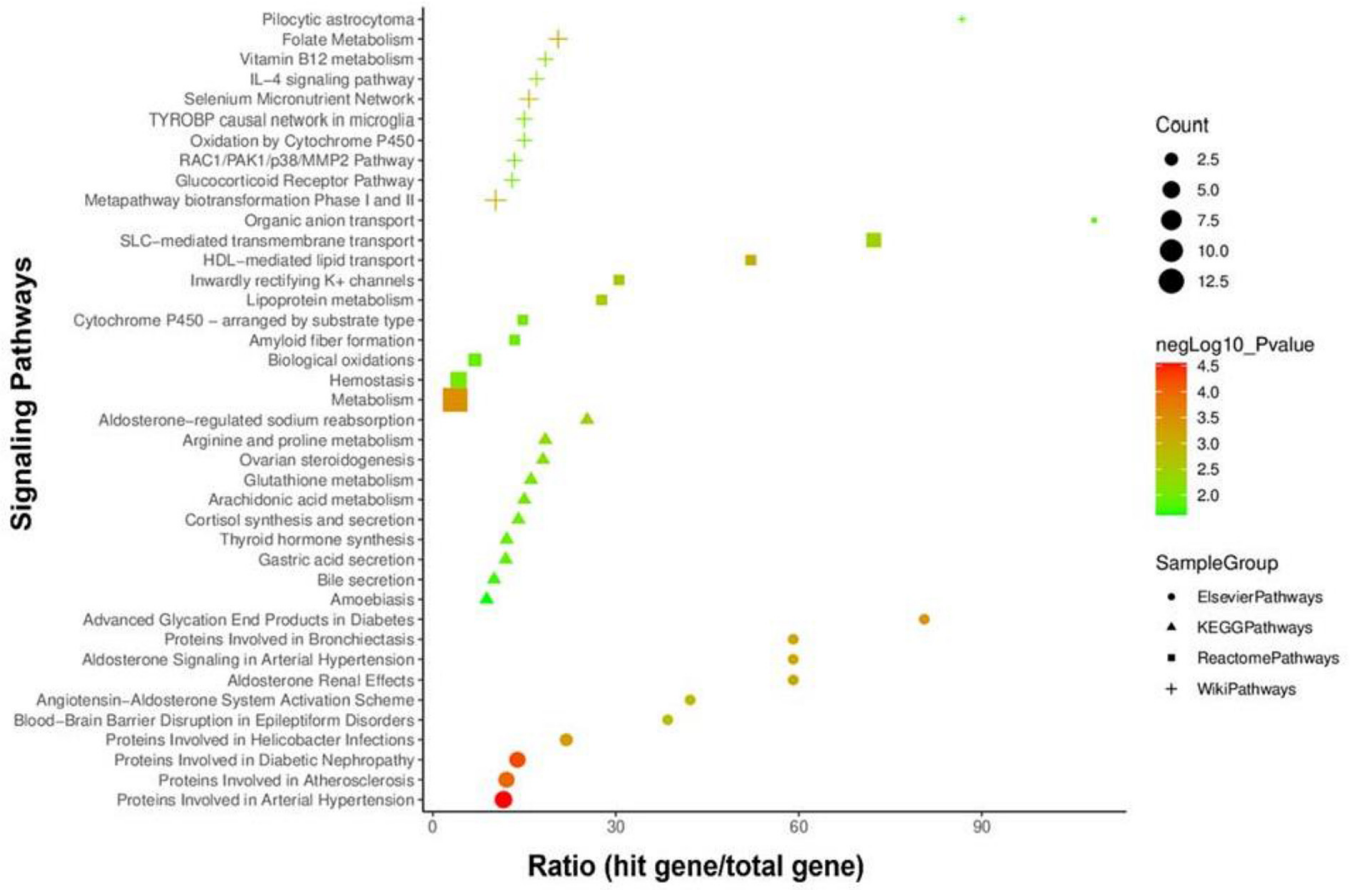
Signalling pathways that are linked to the dysregulated genes in common We utilized the Elsevier collection, Reactome (2016), KEGG (2019), and WikiPathways (2019) datasets to find the most essential pathways, and then provided the top 40 (top-10 from each of the three databases) with an adjusted *P*-value less than 0.05.

The enrichment study analyzes both the GO terms analysis and the KEGG pathway analysis. Following the discovery of unique DEGs linked to RCC infection, a variety of databases were used to identify GO keywords and cell-informative pathways (Wiki, KEGG, Elsevier, Reactome, and the GO). In the course of the complicated diseases, a large number of signaling pathways and GO keywords are involved. We identified critical pathways and gene ontologies that may link RCC and the four diseases under consideration using 47 common DEGs disease. A wide spectrum of signaling pathways and GO terms are involved in the orchestration and progression of diseases in complex disorders. We used 47 common DEGs to identify major pathways and gene ontologies that may link RCC and the four diseases under consideration show 40 significant pathways and 30 GO terms from three datasets (top-10 terms from each; Supplementary Tables S10 and S11). Pathways and GO terms were selected based on the number of genes involved and the *P*-value less than 0.05. The majority of the pathways are linked with metabolism (15), followed by a wide range of metabolic disorders. Other pathways include angiogenesis/cancer (two), brain disease (two) and metabolisms (two) as shown in Figure 5. Top-10 pathways are metabolism (13), proteins involved in arterial hypertension (six), proteins involved in diabetic nephropathy (five), proteins involved in atherosclerosis (five), hemostasis (five), meta pathway biotransformation Phase I and II (four), SLC-mediated transmembrane transport (four), proteins involved in helicobacter infections (three), folate metabolism (three), selenium micronutrient network (three), and biological oxidations (three) (Figure 5).

**Figure 6:**
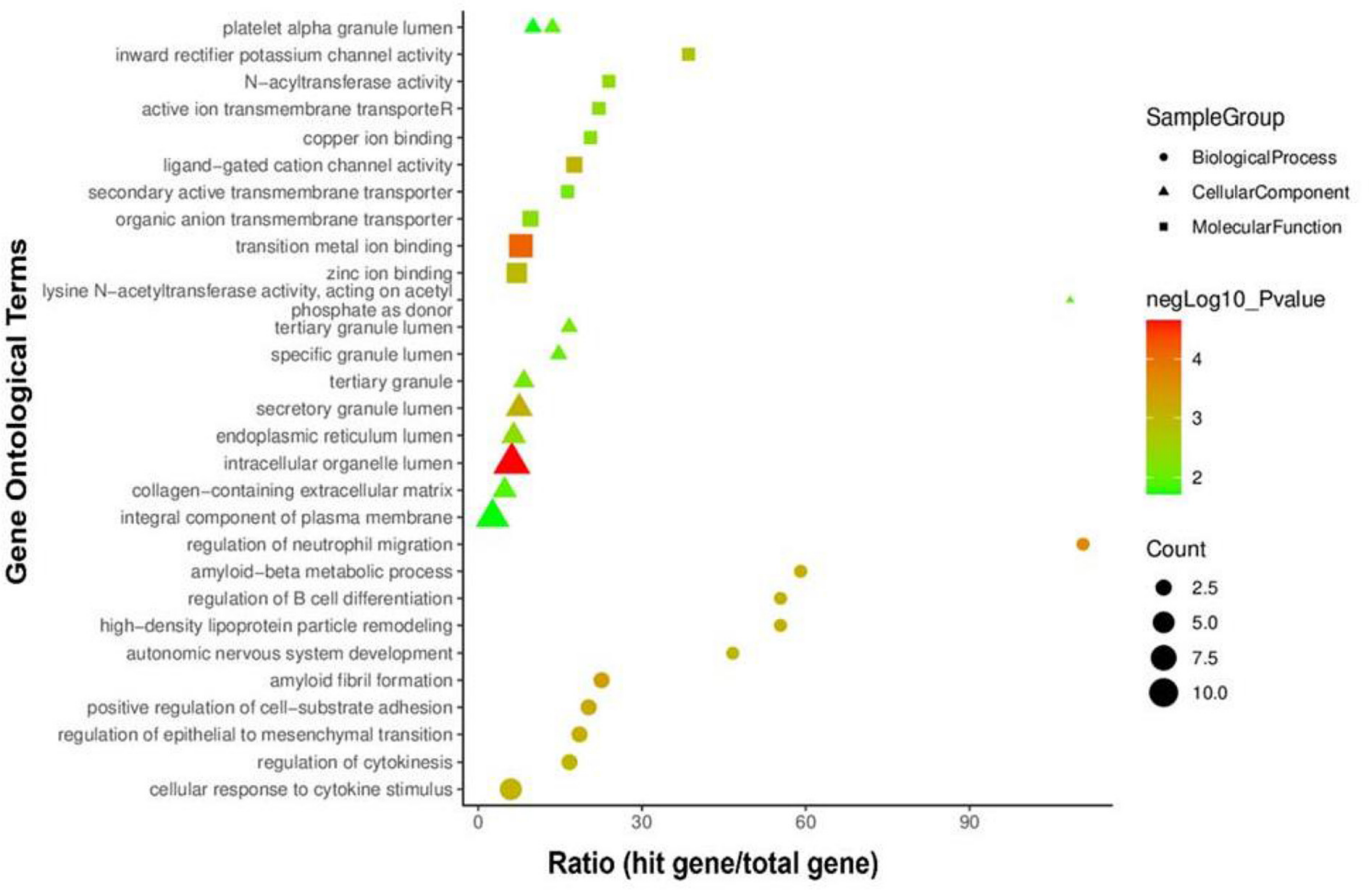
Ontological groupings of genes linked to dysregulated genes in common Biological process (2018), cellular component (2018), and molecular function (2018) datasets were utilized to generate GO words. The top-30 most relevant GO keywords (top-10 from each of three databases) with an adjusted *P*-value less than 0.05 were shown in the graph.

All GO keywords were supposed to include the top ten biological processes, cellular components, and molecular functions (Table 1). Overpresented GO groups were predicted for common DEGs in addition to signaling pathways. Based on the number of genes and a *P*-value of less than 0.05, a total of 30 GO terms were selected. Top-10 GO terms were identified as intracellular organelle lumen (ten), integral component of plasma membrane (eight), transition metal ion binding (seven), cellular response to cytokine stimulus (six), zinc ion binding (five), secretory granule lumen (five), endoplasmic reticulum lumen (four), collagen-containing extracellular matrix (four), amyloid fibril formation (three), and positive regulation of cell-substrate adhesion (three) as shown in Figure 6. These ontological characteristics were seen frequently in RCC complications. As a result, in RCC disease, they could represent risk factors or regulatory checkpoints.

### PPI network analysis of DEGs

**Figure 7:**
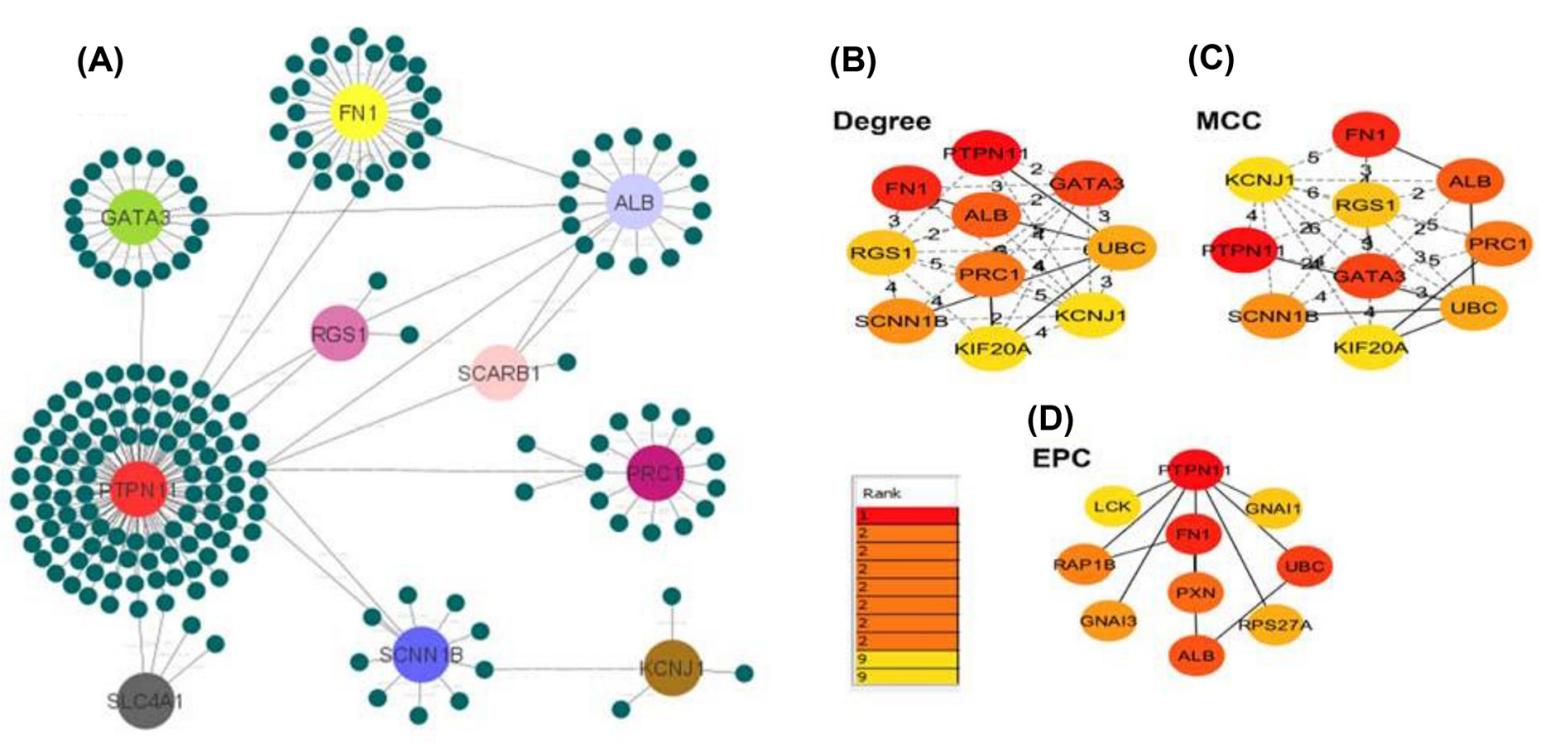
The PPI networks are an illustration here This network comprises (**A**) 185 proteins in total, including 13 common DEGs in which hubs were identified and highlighted using the STRING data-degree base’s technique (confidence cutoff of 900). Hub proteins predicted by (**B**) degree, (**C**) MCC, and (**D**) EPC techniques are depicted in three smaller networks. Top-10 hub nodes are rated with a red-to-yellow-colored gradient for all methods.

The PPI network of DEGs has been built from the common DEGs’ interactions, which has a customized representation with 208 nodes and 216 edges, Figure 7A depicts the PPIs network. Subsequently, the top six hub genes were identified from the network as the hub genes of RCC using the Network Analyst platform and further customized by using the Cytoscape v.3.7.2 software, based on the STRING database (Figure 7B–D). The corresponding PPIs network is primarily concerned with hub gene discovery, module analysis, and prediction of successful drug molecules. Figure 7 depicts the PPIs network, which has a customized representation with 317 nodes and 344 edges. We used four approaches to find the hub proteins, each of which identified the top ten hub nodes in the PPI network (Figure 6B–E). Except for EPC, six out of ten hub proteins were found in all approaches.

We identified six hub nodes as potential hub proteins, namely *PTPN11, FN1, ALB, GATA3, PRC1*, and *SCNN1B*, as predicted by three independent approaches and displaying a minimum of eight interconnections. Table 2 summarizes the biological roles of these hub proteins. On the other hand, the EPC technique predicted just three proteins (*PTPN11, FN1, and ALB*) as hubs from the common DEGs, which were not discovered by other methods.

**Table 2:** The possible hub nodes name, biological function, and role in cancer progression along with UniPort identities

Hub proteins	Full name	Features	UniProt ID	Ref.
PTPN11	Protein tyrosine phosphatase nonreceptor type 11	It activates the Ras-Erk pathway in an abnormal manner. Shp2 is encoded by the human gene Ptpn11, which can either stimulate or prevent tumor growth. In advanced renal cell carcinoma and glioblastoma, mutations in the *PTPN11* gene have been linked to a higher response rate and longer survival.	Q90687	[53–55]
FN1	Fibronectin 1	FN1 is indeed a glycoprotein that plays significant part in cell proliferation and migration in several processes, including embryogenesis, wound repair, blood clotting, host immunity, and metastasis. The expression of the FN1 protein in the cytoplasm of RCC patients is linked to a greater disease-related death rate, suggesting that it may play a role in RCC progression.	P02751	[56]
GATA3	GATA-binding protein 3	GATA3’s key role in T-helper 2 (TH2) cellular development is as a central transcriptional activation. GATA3 is required for nephric duct morphogenesis in the pro-/mesonephric kidney and is critical for renal development. GATA3 might be important in ccRCC.	P23771	[57–59]
ALB	Albumin	One of most prevalent protein in plasma is albumin, and its concentration in the blood is strictly controlled due to its importance in maintaining homeostasis. In individuals with T2DM and DN, a lower serum albumin level was related to lower kidney function and a poor renal prognosis, regardless of clinical or histological characteristics.	P02768	[60,61]
PRC1	Protein regulator of cytokinesis 1	It is a cytokinesis regulator that cross-links antiparallel microtubules. PRC1 is a potential Wnt target that promotes hepatocellular carcinoma cancer proliferation, metastasis, and carcinogenesis.	O43663	[62]
SCNN1B	Sodium channel epithelial 1 β subunit	The amiloride-sensitive *SCNN1B* gene codes for the epithelial sodium channel’s β subunit, which regulates blood pressure. It regulates salt reabsorption in the kidneys, intestines, lungs, and sweat glands.	P51168	[63]

### Transcriptional and post-transcriptional biomarkers

**Figure 8:**
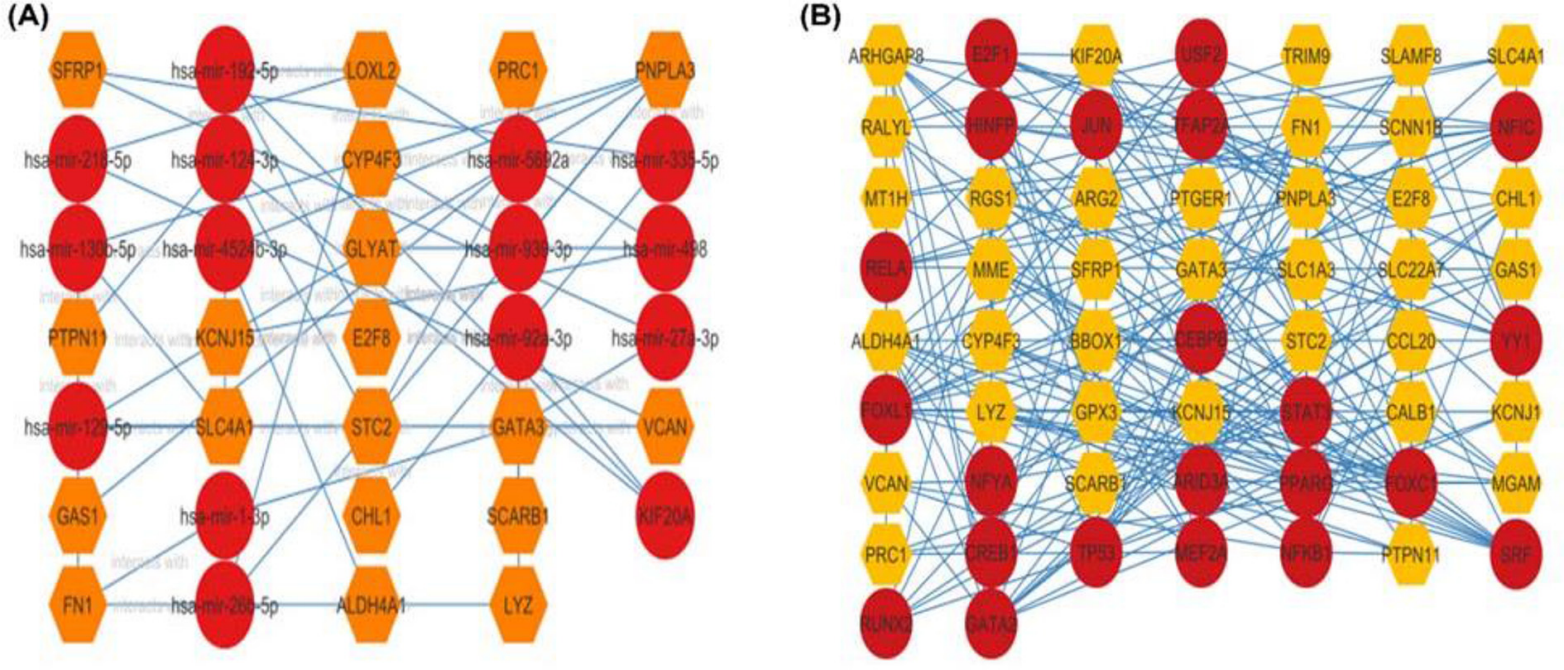
The commonly dysregulated genes were linked to gene regulatory networks The gene-miRNA interacting network (**A**) and gene–TF interacting network (**B**) are depicted in this diagram. Degree centrality less than and equal to 2 and 5, respectively, were used to filter the interaction network of miRNAs and TFs. Hexagons show common DEGs, whereas dark red circles represent associated miRNAs and TFs, respectively, in these networks.

The interaction of TFs and miRNAs with DEGs might be applied to regulate DEG expression. The Network Analyst platform is used to construct the coregulatory system of TF–miRNA interaction, which is then reintroduced in the Cytoscape program for better visualization. Using the common DEGs, we discovered 14 miRNAs and 58 TFs that may impact the expression profile of such genes and contribute to the disease process as shown in Figure 8. Among the miRNAs, we discovered eight miRNAs (i.e., hsa-mir-26b-5p, hsa-mir-335-5p, hsa-mir-92a-3p, hsa-mir-124-3p, hsa-mir-192-5p, hsa-mir-130b-5p, hsa-mir-218-5p, hsa-mir-498) with betweenness centrality ≥ 50 (Figure 8A). These miRNAs may play a role in the development of RCC and other diseases. The top nine TFs (i.e., FOXC1, FOXL1, GATA2, NFIC, YY1, USF2, ALDH4A1, JUN, SRF) were discovered with betweenness centrality ≥ 50 out of a total of 58 TFs as shown in Figure 8B and listed in Supplementary Table S12. Apart from these, as discussed in the Discussion section, our analysis covers TFs and miRNAs, which are critically important in the advancement of RCC and other diseases.

### Predictive drug compounds

**Figure 9:**
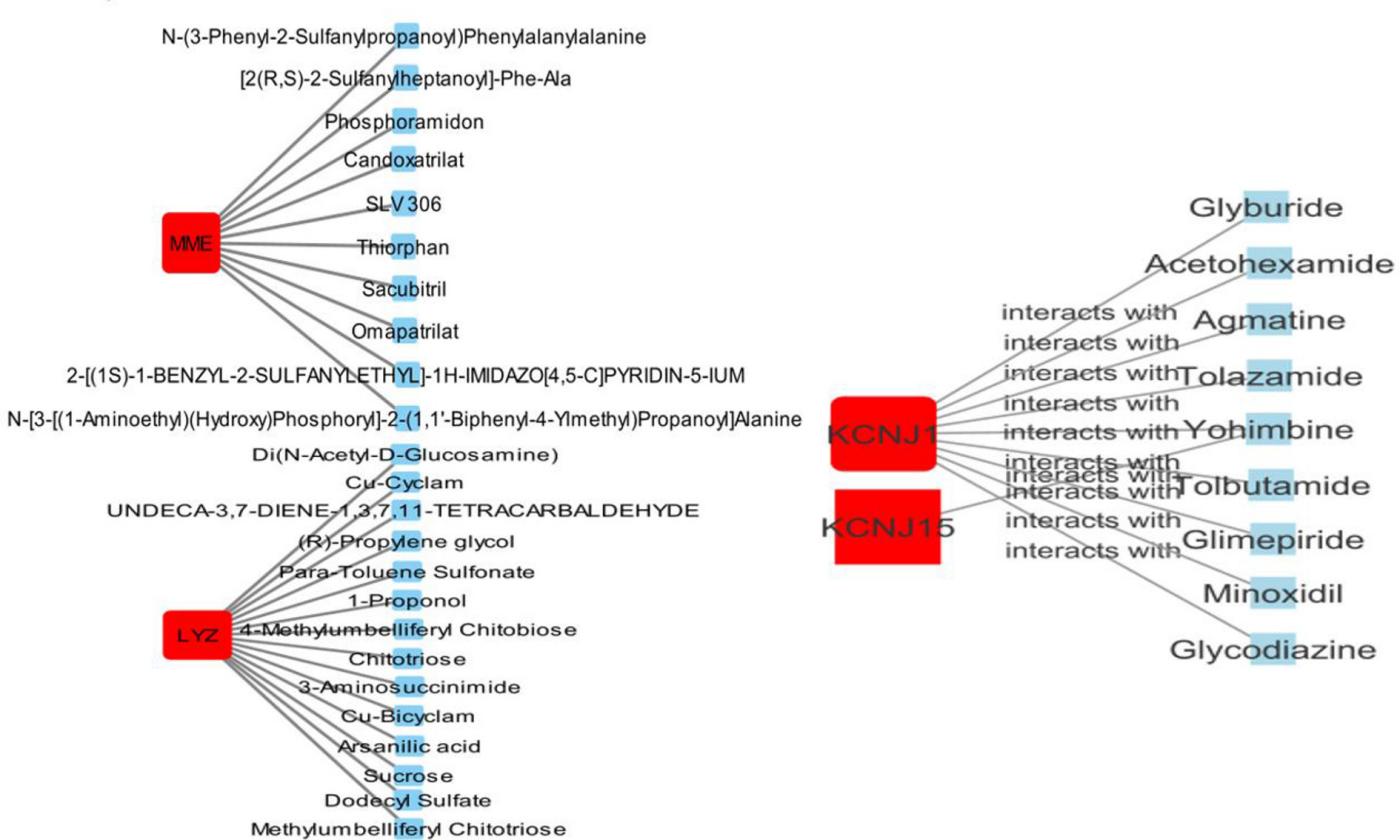
Protein–drug networks are an illustration here PDI is a network of people who work together to solve problems. The common dysregulated genes are shown by cadmium red color, whereas the interacting pharmacological molecules are represented by blue color.

We discovered 35 therapeutic compounds working against four proteins (i.e., LYZ, KCNJ1, KCNJ15, and MME) out of 47 DEGs by using the PDI method. Figure 9 showed the connections between specific proteins and potential drug compounds. In total, 16 drug compounds showed antagonistic connectivity to LYZ, followed by KCNJ15 (nine) and MME (11). Only six drugs (i.e., Bethanidine, Sacubitril, Glyburide, Glimepiride, L-Aspartic Acid, and 1-Propanol) were determined to be approved, while the rest were either experimental (21) or investigational (eight), i.e., Bethanidine, Sacubitril, Glyburide, Glimepiride, L-Aspartic Acid, 1-Propanol. The majority of the compounds, such as Sacubitril, Bethanidine, Glyburide, and Glimepiride, were antagonist drug candidates.

## Discussion

We investigated gene expression profiles and compared them with RCC. Four major comorbidities (i.e., OB, CKD, T2D, and HCV) overlap with RCC pathophysiology that leads to RCC deadly. We discovered that RCC was more relevant to OB, CKD as evidenced through their common expression of the gene in compared with T2D and HCV. Six essential proteins were also discovered, all of which are important in RCC and its comorbidities. Moreover, the study’s common DEGs were discovered to provide an effect on the lives of obese individuals. Later, significant gene regulatory elements including TFs and miRNAs were found, which effectively regulate the primary disease networks.

The RCC transcriptome identified 47 DEGs that are shared by all four comorbidities, according to the comparison study. The majority of genes including 34 out of 47 DEGs were up-regulated, whereas only a minority (13) were down-regulated (i.e., *PTPN11, BIN2, SCARB1, PRC1, SIGLEC1, LOXL2, LYZ, CCL20, FN1, TRIM9, CD52, VCAN*, and *RGS1*). Surprisingly, we discovered that RCC contains the majority of DEGs (28) with OB, 14 DEGs with CKD, and five DEGs with T2D. While the latter scenario is predictable given the pathogenesis location, the connection between the two is significant. This could be co-ordinated by a number of genes working together. For instance, *SLC4A1* a causal gene for distal renal tubular acidosis or RBC abnormalities [64] and CKD has recently been identified in 30–80% of primary dRTA patients [65]. Furthermore, the *SCLC4A1* gene may be a target for RCC therapy [66], as well as *SLC4A1* was found to be strongly linked to blood pressure variations [67]. An additional gene is the *VCAN* gene that has a close correlation to lean body mass [68] and possible genetic biomarkers for HCV–HCC prognosis [69]. *VCAN* is an extracellular matrix component connected to a variety of cancers, and this gene may be used as a treatment or biomarker for RCC [70]. As a result, the findings are consistent with those of the prior study. Furthermore, RCC shares the down-regulation of the *LOXL2* gene with OB and CKD. Overexpression of *LOXL2* or *LOXL3* in epithelial cells causes EMT, indicating that they may play a role in tumor growth. Most interestingly, *LOXL2* was found to be up-regulated in NAFLD people with T2D, and *LOXL2* hepatic and circulatory amounts were linked to the advancement of histological fibrosis [71,72].

Alteration and regulation of genes require knowledge of the precise genomic loci that affect their activity [73]. A number of genomic loci have been linked to the development and progression of cancer in recent studies, revealing genetic abnormalities in RCC [74–76]. Cancer is caused by the accumulation of many mutations that impair the physiology of the genetic material [73]. The most of common DEGs are found on chromosomes 1 and 5. In a mouse model study, chromosome 1 was revealed to be involved in the development of OB [77]. Furthermore, the discovery of a substantial association with a quantitative trait locus on chromosome 5 supports the replication of a previously described quantitative trait gene for OB-related phenotypes [78]. Furthermore, the majority of the genes were discovered to be associated with kidney and liver tissues. As a result, the characteristics of RCC corroborate our findings. We used the common DEGs that showed possible risks and diseases to further validate the link between RCC and chosen comorbidities and other diseases. The Metascape server [41] also recommended the top 20 complications in addition to the identified comorbidities, including renal carcinoma, mammary neoplasms, androgen-insensitivity, mammary carcinoma, fatty liver disease, nonalcoholic steatohepatitis, hypokalemic alkalosis, hypokalemia, cholelithiasis, and renal insufficiency, etc. Surprisingly, new reports corroborate our findings. RCC severity, for example, is common in individuals with neoplasm, accounting for 80–85% of all malignant renal tumors and 2% of all malignancies and related injuries [79]. Furthermore, the DEGs that were discovered were also placed into the Expression Atlas to see how much they were expressed within comorbidities. Like RCC, most of the shared DEGs were found to be substantially up-regulated in a variety of diseases, i.e., Crohn’s disease, glioma, ulcerative colitis, esophageal adenocarcinoma, squamous cell carcinoma, neoplasm, clear cell renal carcinoma, and colon carcinoma. Therefore, this result coincides with the Metascape server’s forecast, validating our research.

We also selected the top 40 pathways which might impact disease progression. Notably, the majority of genes and pathways were discovered, which are associated with metabolic activities. RCC has earned the name ‘metabolic disease’ due to its wide range of metabolic abnormalities and aberrations that result in the development of the tumor’s genetics [80]. All histologists of RCC show improvements in metabolic reactions with disease advancement, which is consistent with Hanahan and Weinberg's metabolic abnormalities that have been identified as a characteristic of cancer [81]. Proteinuria is the most common symptom of diabetic kidney disease (DKD) and is a risk factor for both renal and cardiovascular disease [82]. Specific metabolic intermediates may drive disease progression; for example, alterations in cell surface protein expression have been linked to acetyl-CoA synthetase, which is connected with cell motility and invasion. Due to its characteristics, the PI3K/AKT signaling pathway is necessary for optimal metabolism, and its imbalance leads to OB and types 2 diabetes mellitus [83]. In OB, interleukin-4 and interleukin-13 indicated via the IL-4R modulates adipose tissue lipolysis, insulin sensitivity, and liver fibrosis [84]. Pre-existing metabolic disorders are linked to a higher risk of RCC and vice versa [85]. As a result, these metabolic pathways could be connected to RCC, revealing important pharmacological target checkpoints. Target key proteins or enzymes that are implicated in dysregulated metabolic pathways are being developed for the treatment of RCC, for example, due to the high prevalence of metabolism in RCC. Like pathways, GO terms pathways included the intracellular organelle lumen, plasma membrane, transition metal ion binding, zinc ion binding, secretory granule lumen, cellular response to cytokine stimulus, etc. This may give additional facts about the regulatory network and contribute to RCC pathogenic mechanisms.

The shared DEGs were used to create a PPI network that depicted their interaction and identified the important disorder components (hubs) in RCC and complications. Hub proteins are known as which eight or more connections have, while less than four connections are regarded as nonhubs [86]. Hub proteins are considered functionally relevant because they all have many interacting nodes inside a network [87]. We discovered six common hub proteins (PTPN11, FN1, ALB, GATA3, PRC1, and SCNN1B) involved in renal tubules and RCC risk factors using various approaches. In our research, we discovered that PTPN11 is a hub protein that is involved in numerous forms of leukemia and hepatic carcinogenesis [54]. FN1 is a plasma protein that may be important in more prevalent renal diseases like diabetic nephropathy, IgA nephropathy, and lupus nephritis [88]. GATA3 expression is also a sensitive diagnostic for RCC and CRC [89] as it is a major regulator of nephric duct morphogenesis [90]. PRC1 is a new regulator in the Wnt/-catenin signaling pathway that regulates microtubule organization [91]. The development of RCC is promoted by Wnt/-catenin [92]. As a result, if their functional activity in RCC is confirmed, those hub proteins could be regarded prospective biomarkers or therapeutic targets.

Furthermore, translation rate is regulated by TF [93], whereas miRNA is involved in RNA-silencing and post-transcriptional epigenetics [94]. As a result, both are required to comprehend the progression of a certain disease. The links between shared DEGs, their TFs, and regulatory miRNAs were investigated in the present study. Several TFs were identified, including FOXC1, FOXL1, GATA2, and GATA3, which are known to be related to RCC [95]. Four of the nine miRNAs were connected to OB (i.e., mir-26b-5p, mir-335-5p, mir-124-3p, and mir-130b-5p) [96–98]. For instance, Chartoumpekis et al. demonstrated that the down-regulation of miR-192 is related to the progression of OB in mice [99]. MiR-26b suppresses hepatocellular carcinoma development by adversely regulating ZNRD1 and Wnt/-catenin signaling, providing important insights into the molecular mechanisms of RCC metastasis [100,101]. MiR-124-3p, a tumor-suppressive miRNA that targets CRKL, suppresses HCC carcinogenesis [102]. Adipose miR-130b and miR-17-5p expression in severe patients with CKD were linked to body composition characteristics, frailty, and predicted cardiovascular events and death [103]. Wang et al. discovered miR-130b as a possible biomarker for OB, hypertriacylglycerolemia, and metabolic disease [98]. MiR-335 acts as a connection between inflammation and adipose tissue metabolism, providing important mechanistic insight into the molecular basis of GT activity during OB [104]. Furthermore, certain miRNAs linked to breast cancer (miR-498) and colorectal and non-small cell lung cancer (miR-92a) were discovered, both of which are common in RCC [105–107]. Overall, these results, together with those in the additional file(s), are clinically relevant and may offer insight into the origin and development of RCC, and any novel potential therapeutic approaches.

Finally, several drug possibilities are predicted based on the most common DEGs. Numerous treatments have been revealed to have therapeutic potential on kidney cancer, as expected. For example, Glyburide and Glimepiride are both used to treat diabetes mellitus type 2 [108,109]. Glyburide increases intracellular potassium and calcium ion concentrations via closing ATP-sensitive potassium channels on β cells, which enhance insulin production [110,111]. Insulin and IGFs are potential players to cancer formation and progression, including RCC [112]. Preliminary studies have shown that *in vitro* and *in vivo*, ATP-competitive inhibitors of mTOR decrease the development of RCC cell lines more efficiently [113]. Sacubitril, an approved first-in-class drug that contains a neprilysin (NEP) inhibitor (sacubitril) to treat heart failure, was also discovered [114,115]. The initial symptom of RCC is heart failure [115]. Hence, the discovered therapeutic compounds might be examined to see if they can protect RCC patients. Overall, these data are of clinical interest and may shed light on the cause and progression of RCC, as well as any new prospective therapeutic strategies.

## Conclusion

In the present study, we investigated and analyzed the gene expression profile of RCC with associated four comorbidities to better describe the pathophysiology that is responsible for progressing to RCC. We identified biological domains, regulatory molecules, pathways, and potential biomarkers through the network-based gene expression profile. While this interaction may uncover disease modifying environments as possible drug targets and the signaling pathways may reveal potential molecular checkpoints that are expected to ameliorate the motion of therapeutic development against the RCC progression. The potential six essential hub proteins can be used to create novel diagnostic tools and as drug candidates depend on their function in advancing therapy in RCC cure. Nevertheless, a further experimental investigation is required to determine the viability of using or targeting genetic factors in treating the disease.

## Supplementary Material

Supplementary Tables S1-S12

## Data Availability

All supporting data are included in supplementary files. Raw data associated with the paper are available and can be accessed by contacting the authors

## Abbreviations

CKD: chronic kidney disease
CRKL: CRK-like proto-oncogene, adaptor protein
DEG: differentially expressed gene
dRTA: distal renal tubular acidosis
EMT: Epithelial-mesenchymal transition
EPC: edge percolated component
FC: fold change
FOXC1: forkhead box C1
FOXL1: forkhead box L1
GATA2: GATA-binding protein 2
GATA3: GATA-binding protein 3
GDN: gene-disease network
GO: gene ontology
GSE: gene expression omnibus
HCC: Hepatocellular carcinoma
HCV: hepatitis C virus
KCNJ15: potassium inwardly rectifying channel subfamily J member 15
KEGG: Kyoto Encyclopedia of Genes and Genomes
LOXL2: lysyl oxidase homolog 2
LYZ: lysozyme
MCC: maximal clique centrality
MME: membrane metalloendopeptidase
MT: Mitochondrial
NAFLD: Nonalcoholic fatty liver disease
OB: obesity
PDI: protein–drug interaction
PPI: protein–protein interaction
RCC: renal cell carcinoma
SLC4A1: solute carrier family 4 member 1
T2D: type 2 diabetes
TF: transcription factor
TH2: T-helper 2
VCAN: versican
ZNRD1: zinc ribbon domain-containing 1
